# Supplementary material for: The Vaccine Hesitancy Profiles and Determinants of Seasonal Influenza among Chinese Community Healthcare Workers: A Cross-Sectional Study
Source: Vaccines (Basel). 2022 Sep 16;10(9):1547. doi: 10.3390/vaccines10091547 (PMC9505772; doi:10.3390/vaccines10091547)
Supplement: Supplementary file 1 [file vaccines-10-01547-s001.zip › vaccines-1891892-supplementary.pdf]

Knowledge, Attitudes, and Practice Survey Questionnaire: Community healthcare workers regarding the seasonal influenza vaccine

i. <u>General and Demographic Questions</u>

1. **Name of interviewer**: ______________________
2. **Gender:** ☐ male ☐ female
3. **Date of birth:** ....../……/…………
4. **Area of residence:** ☐ urban ☐rural

5. **What is your education level?**
   a) ☐ Middle school or lower
   b) ☐ High/secondary level or lower
   c) ☐ Junior college
   d) ☐ Bachelor
   e) ☐ Postgraduate or higher

6. **Years of medical service:** ____

7. **What is your highest degree major?**
   a) ☐ Clinical medicine
   b) ☐ Traditional Chinese medicine
   c) ☐ Integrative medicine
   d) ☐ Nursing
   e) ☐ Preventive medicine or public health
   f) ☐ Others
8. **What is your professional qualification?**
   a) ☐ Primary title or lower
   b) ☐ Middle title
   c) ☐ Senior title

ii. <u>Knowledge, attitudes, and practice regarding influenza and influenza vaccine</u>

9. **How about your health condition ?**

   a) ☐ Good
   b) ☐ General
   c) ☐ Fair
   d) ☐ poor

10. **Have you had influenza-like illness (ILI) in the past 3 years ?**

    a) ☐ Yes
    b) ☐ No
    c) ☐ I don’t know

11. **Have you had influenza in the past 3 years ?**

    a) ☐ Yes
    b) ☐ No
    c) ☐ I don’t know

12. Have you asked for leave due to influenza or ILI in the past 3 years?

a) ☐ Yes, about____days
b) ☐ No

13. Did you receive the influenza vaccine in the last flu season?

a) ☐ Yes
b) ☐ No (skip to question 15)

14. How do you pay for influenza vaccination?

a) ☐ Self-paid
b) ☐ Free
c) ☐ Medical insurance

d) ☐ I don't remember

15. Do you plan to receive the influenza vaccine in the next flu season?

a) ☐ Yes
b) ☐ No (skip to question 16)
c） ☐ I don't know (skip to question 16)

16. Why don't you plan to receive the influenza vaccine in the next flu season? (Open -ended question)

a) ☐ Concerned about adverse reactions
b) ☐ Influenza vaccination is unnecessary
c) ☐ Lack of time during working hours
d) ☐ Inconvenience vaccination service
e) ☐ Vaccination fee is not free
f) ☐ Unknown about contraindications of influenza vaccines
g) ☐ Concerned about vaccine efficacy
h) ☐ Doubt about vaccine quality
i) ☐ Others,_______

j) ☐ I don't remember

17. Have you recommended your patients receive the influenza vaccine?

a) ☐ Yes
b) ☐ No
c) ☐ I don't know

18. Why don't you recommend your patients receive the influenza vaccine? (Multiple choice)

a) ☐ Unknown about contraindications of influenza vaccines, and dare not recommend it to patients
b) ☐ Concerned about patients' misunderstanding of selling vaccines
c) ☐ Beyond the scope of HCW's responsibility
d) ☐ Uncertainty of vaccine effectiveness to patients

e) ☐ Patients do not trust the HCW's recommendations on vaccines

19. What do you think is the coverage rate of influenza vaccine for the whole population in China?

a) ☐ Less than 10%
b) ☐ 10%~20%
c) ☐ 20%~30%
d) ☐ Above 30%

20. Which of the following 4 statements is correct ? (Multiple choice)

a) ☐ There is no difference between influenza and cold
b) ☐ The whole population is susceptible to influenza
c) ☐ Immunization is an important strategy for preventing influenza
d) ☐ Influenza virus is transmitted mainly by droplets and aerosols originating from the respiratory secretions of infected people and occasionally via contact with virus-contaminated fomites.

21. As you know, what are the factors that affect the public to receive the flu vaccine ? (Open-ended question)

a) ☐ Unknown about influenza vaccines
b) ☐ Inconvenience vaccination service
c) ☐ Influenza vaccines price is too high to be acceptable
d) ☐ It's unnecessary to receive the influenza vaccine
i) ☐ Others,______

iii. <u>Influenza vaccine hesitancy</u>

22. It's necessary to get an influenza vaccination against influenza ?

a) ☐ Strongly agree
b) ☐ Agree to some extent
c) ☐ Neutral
d) ☐ Disagree to some extent
i) ☐ Strongly disagree

23. The following 9 statements are about seasonal flu and flu vaccine, please state your point of view regarding them.

	**Strongly Agree**	**Agree**	**Neutral**	**Disagree**	**Strongly Disagree**
a. Flu vaccine is effective					
b. Flu vaccine is safe.					
c. I worry about the flu vaccine for vaccine incidents.					

d. I have a high risk of getting flu.					
e. Flu is a big threat to my health.					
f. Flu vaccine is necessary to prevent me from getting flu.					
g. The traffic from my house to the vaccination clinic is convenient.					
h. I can afford the flu vaccine.					
i. I can easily find time to the clinic for flu vaccination.					
